# Refining the role of laparoscopy and laparoscopic ultrasound in the staging of presumed pancreatic head and ampullary tumours

**DOI:** 10.1038/sj.bjc.6602919

**Published:** 2006-01-24

**Authors:** B N J Thomson, R W Parks, D N Redhead, F K S Welsh, K K Madhavan, S J Wigmore, O J Garden

**Affiliations:** 1 Department of Clinical and Surgical Sciences (Surgery), 51 Little France Crescent, Edinburgh, EH16 4SA, UK; 2 Department of Radiology, Royal Infirmary of Edinburgh, 51 Little France Crescent, Edinburgh, EH16 4SA, UK

**Keywords:** pancreatic cancer, laparoscopy, laparoscopic ultrasound, staging

## Abstract

Laparoscopy and laparoscopic ultrasound have been validated previously as staging tools for pancreatic cancer. The aim of this study was to identify if assessment of vascular involvement with abdominal computed tomography (CT) would allow refinement of the selection criteria for laparoscopy and laparoscopic ultrasound (LUS). The details of patients staged with LUS and abdominal CT were obtained from the unit's pancreatic cancer database. A CT grade (O, A-F) of vascular involvement was recorded by a single radiologist. Of 152 patients, who underwent a LUS, 56 (37%) had unresectable disease. Three of 26 (12%) patients with CT grade O, 27 of 88 (31%) patients with CT grade A to D, 17 of 29 (59%) patients with CT grade E and all nine patients with CT grade F were found to have unresectable disease. In all, 24% of patients with tumours <3 cm were found to have unresectable disease. In those patients with tumours considered unresectable, local vascular involvement was found in 56% of patients and vascular involvement with metastatic disease in 17%, while 20% of patients had liver metastases alone and 5% had isolated peritoneal metastases. The remaining patient was deemed unfit for resection. Selective use of laparoscopic ultrasound is indicated in the staging of periampullary tumours with CT grades A to D.

Pancreatic cancer remains an important cause of cancer-related deaths. The majority of patients are unresectable due to liver metastases, peritoneal metastases or local invasion of vascular structures. As the outcome in most patients is poor, accurate staging allows appropriate treatment selection, which usually consists of nonsurgical palliation.

Laparoscopy with the addition of laparoscopic ultrasound has previously been shown to be an effective tool in the staging of pancreatic cancer (John *et al*, 1995) being more predictive of resectability than abdominal computed tomography (John *et al*, 1999). Palliative surgery does not increase the duration of survival compared to endoscopic stenting (Taylor *et al*, 2000), however, operative bypass offers longer palliation of symptoms. If laparoscopic staging identifies unresectable disease, this information can be used to decide the best palliative technique for an individual patient. With the combined use of CT scanning and LUS, unresectable disease is found in 35–54% of patients (John *et al*, 1995; Minnard *et al*, 1998; Nieveen van Dijkum *et al*, 2003).

Some authors advocate helical abdominal CT scanning as the best modality to identify local vascular invasion and metastatic disease and do not perform laparoscopy or laparoscopic ultrasound in the assessment of patients with pancreatic carcinoma (Pisters *et al*, 2001). Loyer *et al* (1996) have described a grading system of vascular involvement based on a dynamic series of 1.5 mm thickness scans in patients with pancreatic adenocarcinoma (Table 1). Along with other groups, they have reported a decreasing rate of resectability with increasing grades of vascular involvement (Loyer *et al*, 1996; Phoa *et al*, 1999; Saldinger *et al*, 2000).

The aim of this study was to identify if assessment of vascular involvement with abdominal CT scanning would allow refinement of the selection criteria for laparoscopic ultrasound in patients presenting with obstructive jaundice from presumed tumours arising from the ampulla or head of the pancreas.

## METHODS

All patients referred to our specialist unit with pancreatic tumours were entered onto a pancreatic cancer database. Patients with tumours of the pancreatic head (including uncinate process) or ampulla who had undergone LUS for staging of their intraabdominal disease were selected for analysis. For inclusion in the study a pancreatic CT scan was required to be available for radiological review. Patients with metastatic disease or peritoneal disease present on CT scanning were excluded from analysis. The selection of all patients with presumed tumours reflects the true nature of patients referred for assessment in our unit where we do not routinely pursue confirmation of a preoperative pathological diagnosis. All patients had a clinical presentation and CT scan appearance consistent with a diagnosis of cancer of the pancreatic head, uncinate process or ampulla.

### CT assessment

The protocol for CT staging of suspected pancreatic tumours involved triple phase CT scanning with 5 mm slices during the arterial phase. During the study period the same spiral CT with a single detector was used for all scans. CT scanning performed prior to referral was only repeated if deemed unsatisfactory by the radiologist.

A single specialist hepatobiliary radiologist who was blinded to the outcome of each patient reviewed the abdominal CT scans. Each tumour was given a CT grade of vascular involvement based on that described by Loyer *et al* (1996). This grading system examines the relationship between the tumour and the major vessels (superior mesenteric vein, portal vein, superior mesenteric artery and hepatic artery). Patients with grade A to D tumours are considered potentially resectable while those with grade E or F tumours are invariably not resectable (Loyer *et al*, 1996). In addition a grade O was added to this grading system for those patients with pancreatic and biliary duct dilatation without the presence of a pancreatic mass.

For analysis, grade A to D tumours where grouped together, as were grade E and F tumours, while grade O tumours were analysed as one entity.

Follow-up was continued until death and/or by outpatient review or General Practitioner contact. Patients were considered to have had benign disease if there had been resolution or no progression of CT scan changes after 12 months of follow-up.

### Laparoscopy and laparoscopic ultrasound assessment

The technique for laparoscopic ultrasound has been previously described (John *et al*, 1999). Laparoscopy was performed under general anaesthesia and if metastatic disease was identified laparoscopic ultrasound was not performed routinely. Routine lymph node examination and biopsy was not performed. Results from all surgeons within the hepatobiliary unit were included. Laparoscopy was performed as an independent procedure and not immediately prior to planned resection.

At laparoscopy an unresectable tumour was determined by the presence of liver or peritoneal metastases as well as visible invasion through the transverse mesocolon.

Tumour size was measured at laparoscopic ultrasound, however, it was not used as an indicator of unresectability. Similarly, invasion of the duodenum, common bile duct or retroperitoneum was not considered to indicate unresectability.

**Figure 1:**
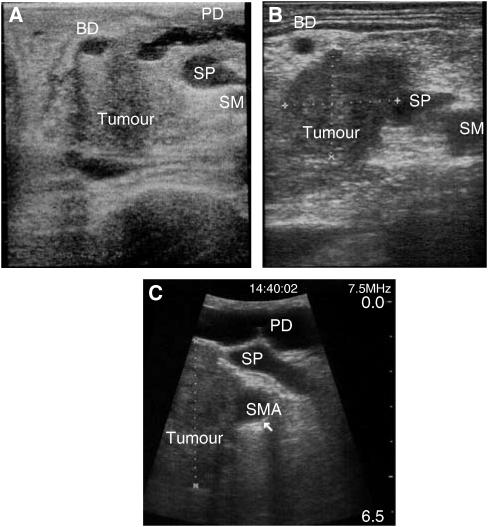
Laparoscopic ultrasound assessment of vascular invasion. (**A**) Resectable tumour free from splenoportal junction. (**B**) Tumour involvement of splenoportal junction. (**C**) Tumour involvement of the superior mesenteric artery (BD – bile duct, PD – pancreatic duct, SP – splenoportal junction, SMA – superior mesenteric artery).

Ultrasonographic assessment of invasion of the superior mesenteric vein, portal vein, superior mesenteric artery or hepatic artery was performed, but particular attention was made of the right lateral aspect of the splenoportal junction. Vascular invasion was indicated if there was (a) obliteration or thrombosis of the vein; (b) a fixed stenosis of the vessel wall; (c) loss of the hyperechoic vessel–tumour interface with encroachment of hypoechoic tumour to the vessel margin; (d) vessel encasement by tumour encirclement and rigidity; (e) presence of tumour within the vessel lumen (John *et al*, 1999) (Figure 1). The accuracy of this technique has previously been validated from this unit (John *et al*, 1995).

Patients in whom definite vascular invasion was not confirmed were still deemed potentially resectable. In general, planned resection of the portal vein was not undertaken, however, involvement of a section of the portal vein at laparotomy was not seen as a contra-indication to resection. Involvement of the superior mesenteric vein, hepatic artery or superior mesenteric artery was considered a contraindication to resection.

## RESULTS

Between April 1995 and March 2002, 564 patients with pancreatic tumours were entered onto the unit cancer database. In all, 154 patients with a CT scan available for radiological grading underwent LUS, however, the procedure was unsuccessful in two patients. In the remaining 152 patients there were 83 (55%) males and 69 (45%) females with a median age of 64 years (range 35–83 years). The eventual pathological diagnosis was pancreatic adenocarcinoma in 93 (61%), presumed pancreatic (but not biopsy proven) in 18 (12%), ampullary cancer in 17 (11%), benign in eight (5%), cholangiocarcinoma in seven (5%), neuroendocrine in five (3%) and four other diagnoses (3%).

**Figure 2:**
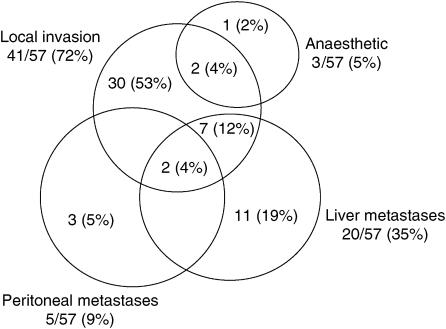
Reason unresectable at laparoscopic ultrasound.

Of the 152 patients who underwent LUS, 56 patients (37%) were deemed unresectable. Table 2 reports the number of patients in each CT grade of vascular involvement and the number with unresectable disease, while Figure 2 illustrates the reason for unresectability. In all, 12% of patients with CT grade O, 31% of CT grade A to D, 59% of CT grade E and all CT grade F patients were found to have unresectable disease.

A total of 96 patients were deemed potentially resectable at laparoscopic ultrasound and their management is detailed in Table 3. Nine patients (9%) received nonsurgical palliation due to patient choice or the development of an intercurrent illness. Resection was possible in 62 (71%) of the remaining 87 patients, with 25 found to be unresectable at laparotomy.

Twelve CT grade E patients were deemed potentially resectable. Ten of these patients underwent a trial dissection, but only three patients had a resectable tumour. All three patients (aged 46, 59 and 63 years) had no evidence of comorbid disease and of these, one had a portal vein resection for a neuroendocrine tumour and is alive with metastatic disease 5 years post resection. The two remaining patients had pancreatic adenocarcinoma and one is alive 19 months after surgery while the other died 16 months post resection.

Of the pancreatic adenocarcinomas, 53 of 111 patients (48%) were found to have unresectable disease by LUS, while only two of 17 patients (11%) with ampullary tumours were unresectable at laparoscopic ultrasound.

**Figure 3:**
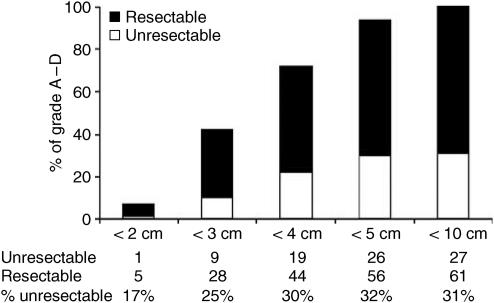
Tumour size on CT for grades A–D and the proportion resectable (88 patients).

The median size of all tumours on CT scanning was 3.0 cm (range 0.0–10.0 cm). Five of 31 (16%) patients with tumours smaller than 2.0 cm were thought to be unresectable at LUS while 51 of 121 (42%) patients with tumours 2.0 cm or larger were deemed to have unresectable disease. In those 88 patients with grade A to D tumours, six patients had tumours smaller than 2.0 cm in diameter and only one (17%) patient was considered unresectable at laparoscopy and LUS. Nine of 37 (24%) patients with tumours smaller than 3.0 cm in diameter were deemed unresectable. Figure 3 details the size of tumours for patients with CT grades A to D as well as the proportion resectable by LUS.

## DISCUSSION

Carcinoma of the head of the pancreas is the sixth commonest cause of cancer related deaths in the United Kingdom. Despite improvements in surgery, chemotherapy and radiotherapy the outlook remains poor with a 1-year survival of only 10% (Office for National Statistics, 1999). In our experience with pancreatic cancer, the median survival in unresectable patients is only 6 months (Engelken *et al*, 2003), however, this increases to 24 months for resectable tumours (Welsh *et al*, 2004). Survival to 5 years does not indicate cure since half of these patients will subsequently die from recurrence of pancreatic cancer (Conlon *et al*, 1996).

If metastatic disease or unresectable local vascular invasion is detected during staging then jaundice can be palliated by endoscopic or radiological biliary stenting (Speer *et al*, 1987). In our own specialist experience, survival is similar following surgical bypass or biliary stenting for the relief of jaundice (Engelken *et al*, 2003) and this is comparable with the findings of a meta-analysis of three randomised control trials (Taylor *et al*, 2000). For patients with unresectable disease, the presence of distant metastases or advanced local disease may alter the palliative options. The detection of liver or peritoneal metastases, or advanced local disease during LUS therefore aids in the choice of palliative therapy for each patient.

Accurate staging of pancreatic cancer allows selection of optimal treatment strategies. In the absence of metastatic disease on abdominal CT scan, 37% of patients considered for resection in our series had unresectable disease at laparoscopic ultrasonography. The majority of these patients were treated with nonsurgical palliation and therefore avoided an unnecessary laparotomy. Cost analysis of the radiological staging of pancreatic cancer has shown that strategies that employ abdominal CT with laparoscopy or laparoscopic ultrasound were consistently less expensive than other strategies, including CT alone (McMahon *et al*, 2001) with the lower cost resulting predominantly from savings made by avoiding an unnecessary laparotomy.

Endoscopic ultrasound had also been reported to be a useful tool in the staging of pancreatic cancer, being more sensitive in determining vascular involvement than abdominal CT scanning (Tierney *et al*, 2001) or angiography (Ahmed *et al*, 2001). However, the limitation of endoscopic ultrasound is that it is operator-dependant and cannot detect small volume peritoneal disease or small subcapsular liver metastases that can be visualised during laparoscopic ultrasound. Although laparoscopic ultrasound is also a highly operator dependant staging tool, we have previously verified the laparoscopic findings by confirmation with laparotomy, laparoscopic biopsy or angiography, to give a sensitivity for unresectable disease of 92% and specificity of 88% (John *et al*, 1995).

Proponents of fine slice helical CT scanning report resection rates of 80% following CT staging (Pisters *et al*, 2001). However, in this report portal vein resection was regularly undertaken and therefore the potential benefit of assessment of vascular invasion by laparoscopic ultrasonography would have been minimised. Other authors believe that open surgical palliation has considerable advantage over endoscopic palliation (Nieveen van Dijkum *et al*, 2003) and therefore regard LUS as conferring little additional benefit. Since portal vein resection is not performed routinely in our unit, nonoperative palliation is preferred and laparoscopic ultrasound remains an important staging tool.

In the report of Loyer and co-workers, there was an increased likelihood of detection of unresectable disease with increasing CT grades of vascular involvement (Loyer *et al*, 1996). All of the 22 patients with grade A and B tumours were resectable with only one patient requiring venous resection. Eight of the nine (88%) patients with grade C tumours were resectable with three requiring venous resection. Eight of 15 patients (53%) with grade D tumours were resectable with six requiring venous resection. No tumours in grade E and F were resected with a negative margin. This study confirms the finding of decreasing likelihood of resection with increasing grade of vascular involvement. The difference in the resectability rates in this series compared to those described by Loyer and co-workers is explained by the addition of a grade O and the low rate of portal vein resection in this series.

Only three of 26 (12%) patients with grade O tumours were deemed unresectable at laparoscopic ultrasound and therefore, in this subgroup of patients, routine laparoscopic ultrasound is not justified. Previous authors have found all patients with grade F to be unresectable (Loyer *et al*, 1996) and this was confirmed in the present study. Therefore, there is clearly no benefit in undertaking laparoscopic ultrasound in those patients with vascular occlusion on CT scan. Tumour size has been suggested as an indicator for predicting patients with a higher risk of metastatic disease (Pisters *et al*, 2001), however, we did not find tumour size to be useful in selecting patients for laparoscopic ultrasound.

Almost one in three patients with grade A to D tumours avoided an unnecessary laparotomy following laparoscopic ultrasound. Most patients with grade E tumours were not resected and therefore laparoscopic ultrasound should be reserved for the staging of young fit patients who are keen to undergo surgery despite the low chance of resection, and in whom nonsurgical palliation is being considered.

Patients who present with gastric outlet obstruction and CT imaging demonstrating a potentially resectable tumour should undergo laparotomy as a hepaticojejunostomy and gastroenterostomy can be performed readily if the tumour is unresectable. If CT scanning shows unresectable disease, consideration should be given to performing laparoscopic gastroenterostomy and endoscopic biliary stenting.

Of those patients deemed to be resectable at laparoscopic ultrasound, approximately a quarter were shown consequently to have unresectable disease. The most likely reason for tumour unresectability in this series was local vascular involvement. Some of these patients were found at laparoscopic ultrasound to have tumour close to the vessels and were given the chance of a trial dissection. Arterial vascular involvement is a contraindication to resection, however, some authors have reported satisfactory results for portal vein resection (Kawada *et al*, 2002).

Further technological improvements in CT, MRI (Ishiguchi *et al*, 2001) and positron emission tomography (Mertz *et al*, 2000), as well as the use of biochemical markers such as C-reactive protein (Engelken *et al*, 2003; Falconer *et al*, 1995) may further refine the indications for laparoscopic ultrasound.

The assessment of vascular involvement with abdominal good quality CT scanning has allowed better definition of the indications for laparoscopic ultrasound. Laparoscopic ultrasound should continue to be used for patients with grade A to D tumours and selected patients with grade E tumours. However, it is not indicated for those patients with no evident mass lesion (grade O) or grade F tumours (vascular occlusion).

## Figures and Tables

**Table 1:** Definition of vascular involvement assessed by CT scanning

**Grade of resectability**	**Definition**
*Grade O*	*Biliary and pancreatic duct dilatation without a mass present (addition)*
Grade A	Fat plane separates the tumour and/or the normal pancreatic parenchyma from adjacent vessels
Grade B	Normal parenchyma separates the hypodense tumour from adjacent vessels
Grade C	Hypodense tumour is inseparable from adjacent vessels, and the points of contact form a convexity against the vessels
Grade D	Hypodense tumour is inseparable from adjacent vessels, and the points of contact form a concavity against the vessels or partially encircle the vessels
Grade E	Hypodense tumour encircles adjacent vessels, and no fat plane is identifiable between the tumour and the vessels
Grade F	Tumour occludes the vessels

Based on the classification of vascular involvement described by Loyer *et al* (1996).

**Table 2:** Detection of unresectable disease at laparoscopic ultrasound based on CT grade

	**CT potentially resectable**					**CT unresectable**	
**Grade**	**O**	**A**	**B**	**C**	**D**	**E**	**F**
Number	26	32	6	11	39	29	9
Unresectable at laparoscopic ultrasound^a^	3	8	1	4	14	17	9
Percentage deemed unresectable	12%	25%	17%	36%	36%	59%	100%

a Liver metastases, peritoneal metastases or vascular invasion.

**Table 3:** Outcome of 96 patients considered to be resectable at laparoscopic ultrasound

**Outcome**	***n* (%)**	**Reason**	***n***
Resection	62 (65%)	Pancreaticoduodenectomy	61
		Local excision	1
Nonsurgical palliation	9 (9%)		9
Palliative surgery	25 (26%)	Missed local invasion	14
		Missed liver metastases	6
		Missed peritoneal disease	1
		Extensive nodal involvement	2
		Missed liver met and local invasion	2
			
Total	96		96
